# Managing sepsis and septic shock in an endothelial glycocalyx-friendly way: from the viewpoint of surviving sepsis campaign guidelines

**DOI:** 10.1186/s13613-024-01301-6

**Published:** 2024-04-24

**Authors:** Toshiaki Iba, Cheryl L. Maier, Julie Helms, Ricard Ferrer, Jecko Thachil, Jerrold H. Levy

**Affiliations:** 1 Department of Emergency and Disaster Medicine, Juntendo University Graduate School of Medicine, 2-1-1 Hongo Bunkyo-Ku, Tokyo, 113-8421 Japan; 2 Department of Pathology and Laboratory Medicine, Emory University School of Medicine, Atlanta, GA USA; 3 Strasbourg University Hospital, Medical Intensive Care Unit-NHC, INSERM (French National Institute of Health and Medical Research), UMR 1260, Regenerative Nanomedicine (RNM), FMTS, Strasbourg University (UNISTRA), Strasbourg, France; 4 Intensive Care Department, Hospital Universitari Vall d’Hebron Universitat Autònoma de Barcelona, Barcelona, Spain; 5 Department of Haematology, Manchester University Hospitals, Oxford Road, Manchester, UK; 6 Department of Anesthesiology, Critical Care, and Surgery, Duke University School of Medicine, Durham, NC USA

**Keywords:** Glycocalyx, Endothelial cell, Sepsis, Shock, Syndecan, Microvasculature

## Abstract

Maintaining tissue perfusion in sepsis depends on vascular integrity provided by the endothelial glycocalyx, the critical layer covering the luminal surface of blood vessels. The glycocalyx is composed of proteoglycans, glycosaminoglycans, and functional plasma proteins that are critical for antithrombogenicity, regulating tone, controlling permeability, and reducing endothelial interactions with leukocytes and platelets. Degradation of the glycocalyx in sepsis is substantial due to thromboinflammation, and treatments for sepsis and septic shock may exacerbate endotheliopathy via additional glycocalyx injury. As a result, therapeutic strategies aimed at preserving glycocalyx integrity should be considered, including modifications in fluid volume resuscitation, minimizing catecholamine use, controlling hyperglycemia, and potential use of corticosteroids and anticoagulants. In this review, we explore treatment strategies aligned with the recommendations outlined in the Surviving Sepsis Campaign Guidelines 2021 with a special emphasis on evidence regarding glycocalyx protection.

## Background

Sepsis is a critical condition complicated by infection-induced organ dysfunction [1]. Tissue malcirculation, resulting from a dysregulated host immune response, is considered the direct mechanism underlying organ dysfunction in septic patients [2]. Microvasculature dysfunction in sepsis includes vasoplegia, loss of endothelial antithrombogenicity, upregulated vascular-blood cell interactions, and increased permeability, all of which play pivotal roles in disease progression [3]. Recent studies report that microcirculatory damage and endotheliopathy in sepsis are triggered by injury to the endothelial glycocalyx [4], and preservation of the glycocalyx and its functions are important for maintaining organ function [5]. Although the current global Surviving Sepsis Campaign Guidelines 2021 (SSCG 2021) [6] do not address endotheliopathy, some recommendations align with glycocalyx protection strategies [7]. In this review, we introduce strategies for glycocalyx protection as they relate to the SSCG 2021. We hope future guidelines will propose recommendations specifically aimed at maintaining glycocalyx integrity.

## The structure and function of the glycocalyx

**Fig. 1:**
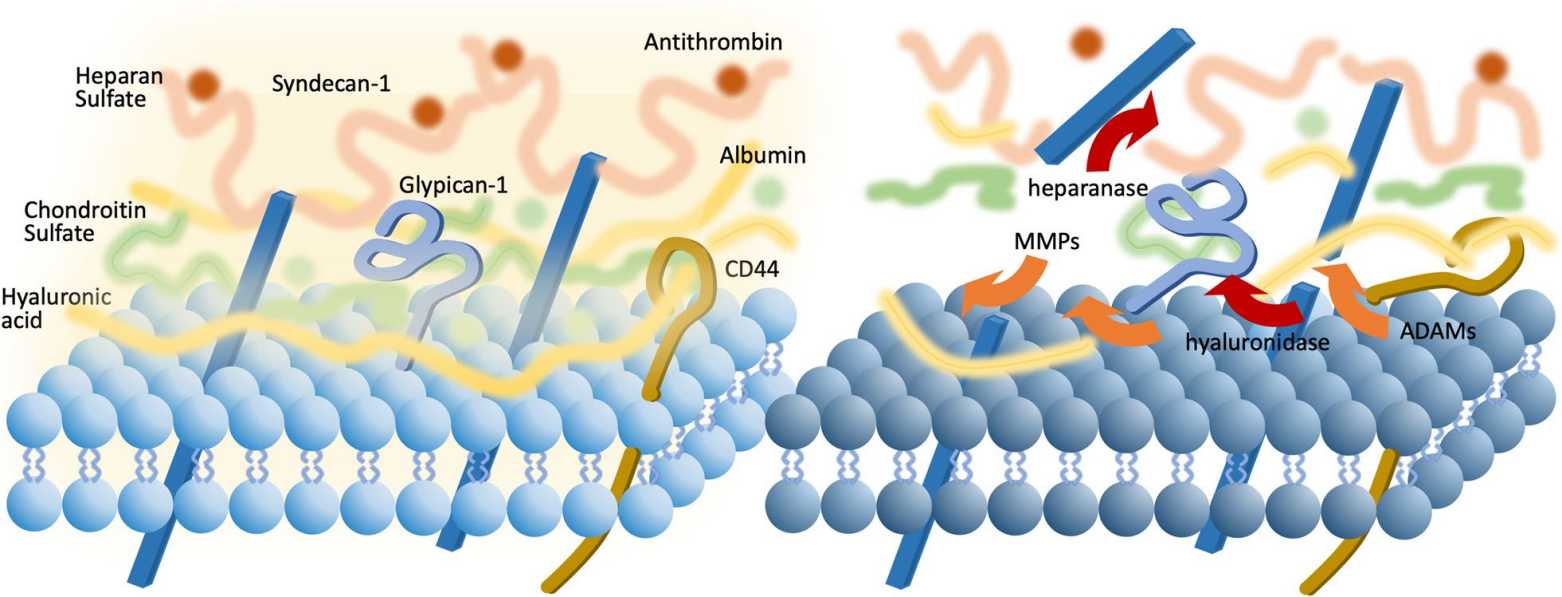
The structure and the degradation of the endothelial glycocalyx. The vascular endothelial glycocalyx comprises proteoglycans and glycoproteins conjugated with highly sulfated glycosaminoglycan side-chains, including heparan sulfate and chondroitin sulfate, as well as the high‐molecular‐weight polysaccharide hyaluronan (left). During sepsis, both enzymatic and non-enzymatic pathways contribute to the degradation of glycocalyx. However, the precise dynamics between proteoglycan cleavage and glycosaminoglycan degradation are only partially understood. Syndecans, glipicans, and CD44 undergo ectodomain cleavage primarily by matrix metalloproteinases (MMPs) and members of the A Disintegrin and Metalloproteinase (ADAMs) enzyme family. In addition to oxygen free radicals, enzymes like heparanases and hyaluronidases play roles in the degradation of heparan sulfate, chondroitin sulfate, and hyaluronan (right)

The endothelial glycocalyx is a carbohydrate-rich gel-like layer up to 3 μm thick that covers the entire luminal surface of the vasculature. It consists largely of membrane-binding proteoglycans (e.g., syndecan and glypican), highly sulfated secreted glycosaminoglycan side-chains (e.g., heparan sulfate and chondroitin sulfate), and high‐molecular‐weight polysaccharide hyaluronan [8]. Hydrophilic polysaccharides retain large amounts of water and bind physiological plasma proteins like albumin and antithrombin [9] (Fig. 1). The glycocalyx modulates flow resistance, vascular permeability, vascular tone, antithrombogenicity, and inflammatory responses. Among them, the regulatory function of vascular permeability has arguably had the strongest impact on conventional thinking. The endothelial glycocalyx represents a critical factor influencing hydrostatic and oncotic pressure differentials between the capillary interior and the interstitium [10]. The recognition of the glycocalyx resulted in the revision of the original “Starling principle,” which does not account for the reduced fluid extravasation from the glycocalyx, and a revised Starling model provides a better explanation of fluid transvascular regulation and responses to fluid resuscitation [11, 12]. Plasma proteins bound to the hydrophilic polysaccharide of the glycocalyx contribute to its stability, and albumin is a major plasma colloid determinant of osmotic pressure. Albumin also transports sphingosine-1-phosphate (S1P), a free radical scavenger, to provide protective effects to the glycocalyx [13] and also functions as a mechanosensor, transducing shear stress and inducing nitric oxide synthesis [14]. As a critical endothelial component, the glycocalyx modulates blood cell-endothelial interactions that include leukocyte rolling, adhesion, and extravasation. The structural architecture and negative charge of the glycocalyx prevent macromolecules larger than 70 kDa and cationic molecules from binding and escaping the vasculature, thereby hindering pathogen extravasation, including bacteria and viruses [15]. In intact endothelial cells, angiopoietin-1 predominantly binds to Tie2, maintaining vascular tone and glycocalyx structure and promoting a quiescent state in healthy vessels. However, in sepsis, the angiopoietin-1/angiopoietin-2 ratio shifts towards Tie2 deactivation, facilitating degradation of the glycocalyx through the release of heparanase and cleavage of the CD44 ectodomain, an anchor for hyaluronan [5].

## Glycocalyx damage in sepsis

**Fig. 2:**
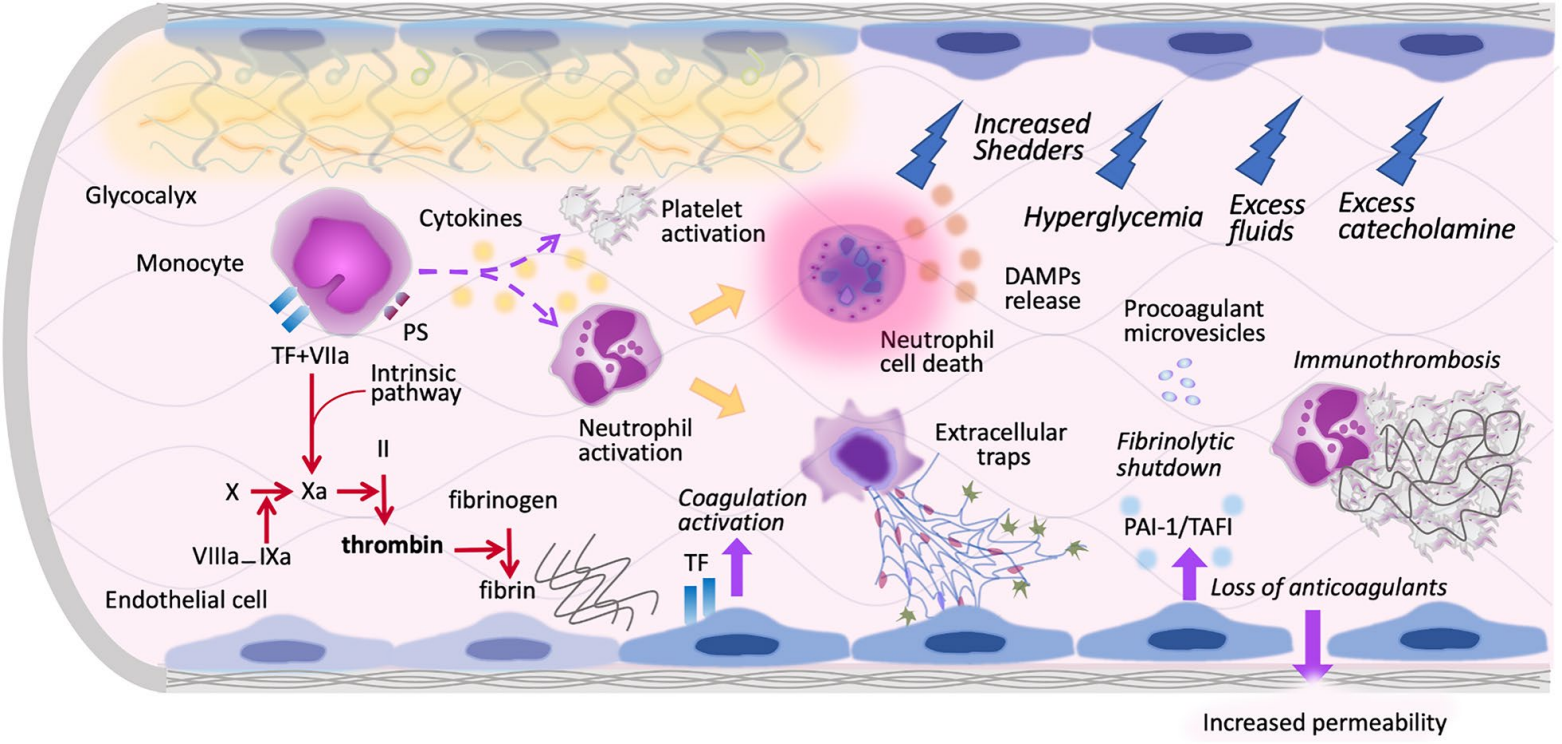
The degradation of endothelial glycocalyx in sepsis. Activated monocytes initiate both extrinsic and intrinsic coagulation pathways by expressing tissue factor (TF) and phosphatidylserine (PS). Simultaneously, neutrophils are activated by proinflammatory cytokines and ultimately undergo cell death, leading to extracellular trap formation. Intravascular thromboinflammation results in endothelial activation, leading to hypofibrinolysis due to increased production of plasminogen activator inhibitor 1 (PAI-1) and thrombin-activatable fibrinolysis inhibitor (TAFI). Thromboinflammation, increased shedders, and hyperglycemia in sepsis also damage the glycocalyx. Moreover, treatments for sepsis and septic shock, such as volume overload and excessive catecholamine administration, further exacerbate glycocalyx damage

**Fig. 3:**
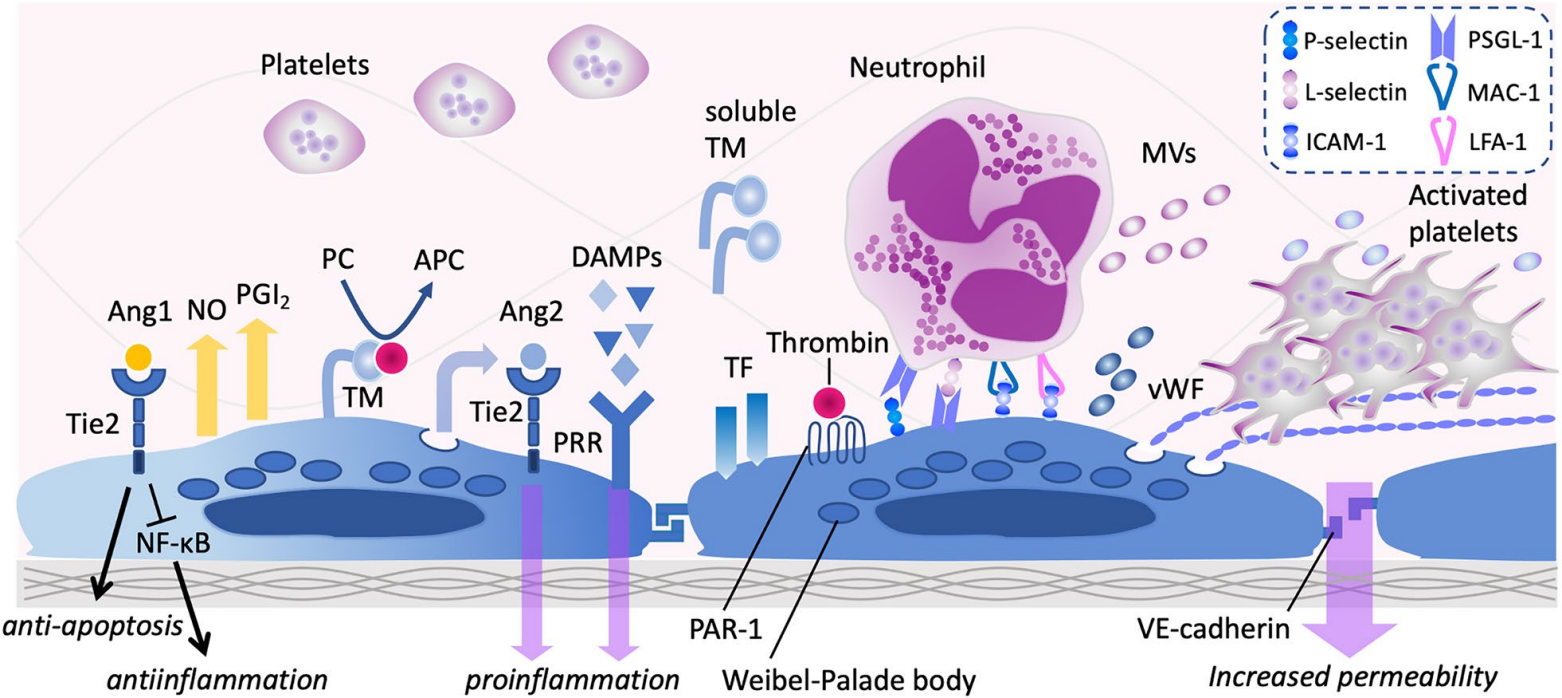
The property of endothelial cells after glycocalyx denudation in sepsis. Intact vascular endothelial cells maintain their antithrombogenicity through various mechanisms, including the production of nitric oxide (NO), prostacyclin (prostaglandin I_2_, PG I_2_), secretion of angiopoietin-1 (Ang1), and expression of thrombomodulin (TM), which facilitates the conversion of protein C (PC) to its active form, activated protein C (APC), in the presence of thrombin. However, in sepsis, the endothelium's antithrombotic properties are compromised. This shift occurs due to the expression of tissue factor (TF), the release of angiopoietin-2 (Ang2), and disruption of the glycocalyx. Thromboinflammation is further exacerbated by the binding of damage-associated molecular patterns (DAMPs) to pattern-recognition receptors (PRRs), thrombin interaction with protease activator receptor 1 (PAR-1), and upregulation of various adhesion molecules. Additionally, the release of von Willebrand factor (vWF) from endothelial cells promotes platelet aggregation. NF*-*κB: nuclear factor*-*κB, MVs: microvesicles, ICAM-1: intercellular cell adhesion molecule-1, PSGL-1:P-selectin glycoprotein ligand-1, Macrophage 1 antigen: MAC-1: LFA-1: lymphocyte function-associated antigen-1

Since the glycocalyx is highly fragile and susceptible to damage during inflammatory responses, increased enzymatic and non-enzymatic proteolytic processes activated in sepsis can cause further injury. Involved mediators include matrix metalloproteinases (MMPs), heparanase, hyaluronidase, thrombin, elastase, and reactive oxygen species [16] (Fig. 2). Nieuwdorp et al. [17] demonstrated that low doses of endotoxin decreased the glycocalyx thickness from 0.6 µm to 0.3 µm in human volunteers. Intravascular inflammation is closely associated with glycocalyx degradation, leading to increased vascular permeability, edema, acquired thrombophilia, and neutrophil and platelet tethering and adhesion [8]. The derangement of the glycocalyx is associated with organ dysfunction and high mortality in sepsis [18]. A disrupted glycocalyx results in capillary leak syndrome (CLS) in sepsis, manifesting as an excessive fluid shift from the intravascular to the extravascular space and resulting in intravascular hypovolemia, interstitial edema, and tissue hypoperfusion [19]. Disruption of the glycocalyx also exposes adhesion molecules such as intercellular adhesion molecule-1 (ICAM-1), vascular cell adhesion molecule-1 (VCAM-1), and P-selectin (Fig. 3). These structural changes foster blood cell adhesion and upregulate the crosstalk between inflammatory cells and coagulation. Enhanced interactions between platelets and leukocytes induce the formation of neutrophil extracellular traps (NETs), further increasing endothelial injury and facilitating thromboinflammation [20].

**Fig. 4:**
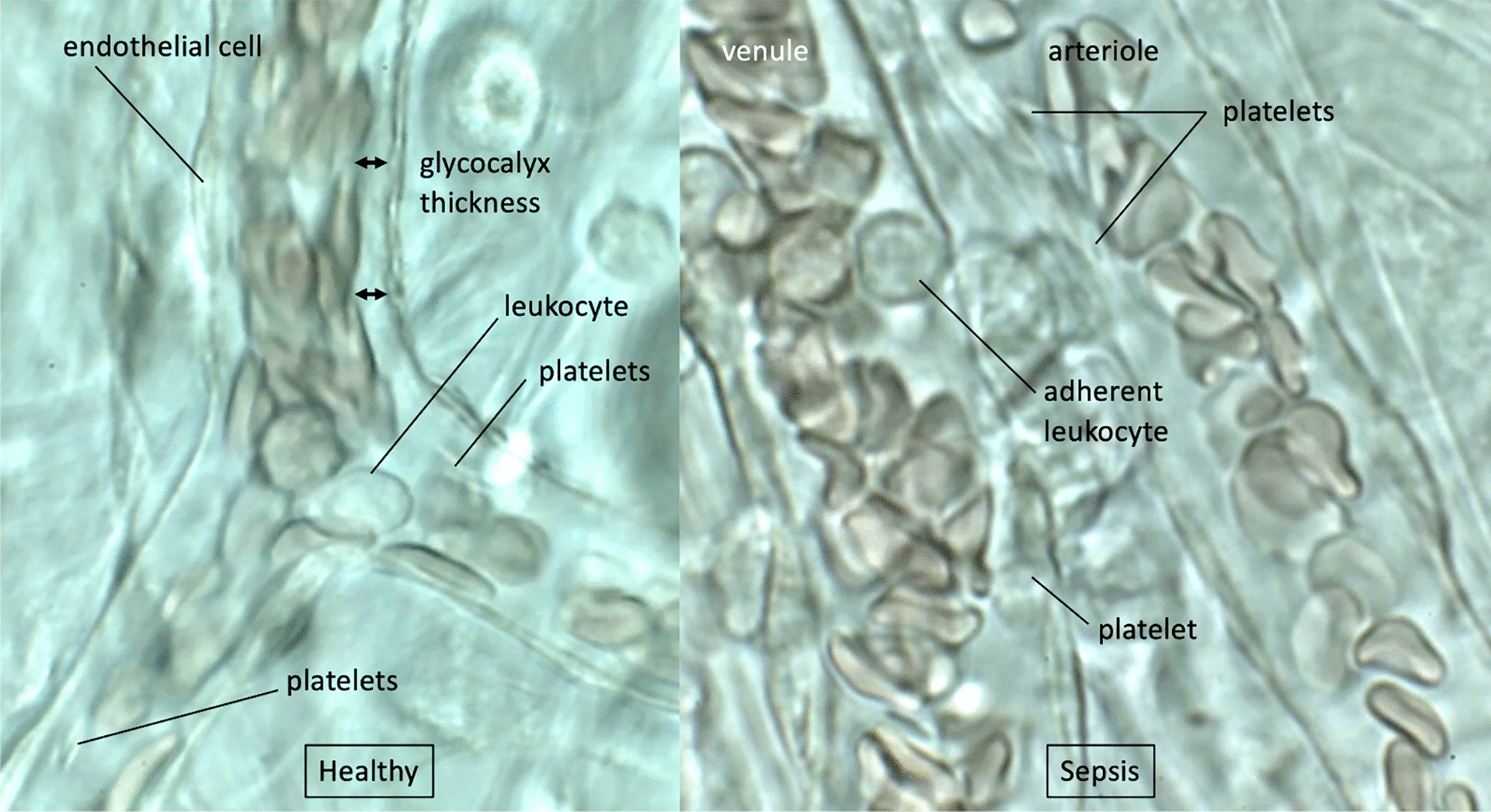
Intravital microscopic view of the glycocalyx. The mesenteric venule was observed under the microscope. The glycocalyx layer is expressed as the gap between endothelial cells and the red cell column in healthy rats. In this view, approximately a 3 μm gap was observed between platelets and endothelial cells. Oval shape (deformable) leukocyte hit the opposite wall and the gap was not certain (left panel). Meanwhile, in the mesenteric vein in rats treated with lipopolysaccharide, round, stiff (undeformable) neutrophil adheres to the endothelium. Platelets also directly attach to the endothelial cell of the arteriole (right panel). The blood flow was decreased after the treatment with lipopolysaccharide

Estimating endothelial injury using intravital microscopy of the microcirculation is reported in experimental models, yet obtaining a clear and well-defined view in patients proves challenging (Fig. 4). Currently, there are two clinically applicable measures for the evaluation of glycocalyx damage: (i) measurement of circulating degraded glycocalyx components, such as syndecan, heparan sulfate, and hyaluronan [21, 22], and (ii) intravital microscopic observation. The real-time evaluation of the glycocalyx is challenging, but sublingual microcirculatory blood flow observation using a side-stream dark field video microscope with analytic software (GlycoCheck^®^) allows for the detection of glycocalyx damage in patients [23]. Unraveling the relationship between glycocalyx damage and microvascular impairment, along with their prognostic and therapeutic significance, is anticipated to offer valuable insights for the development of novel therapeutic approaches for sepsis.

## Glycocalyx damage and capillary leak syndrome

The simultaneous presence of systemic edema, hypovolemia, and hemodynamic instability in various critically ill patients is referred to as CLS. CLS is known to be associated with delayed extubation, longer time in the ICU, and a higher chance of prolonged dependence on vasoactive medication [19]. To date, there is neither an established definition nor standardized diagnostic criteria for this condition. Wollborn et al. [19] aimed to identify common characteristics of CLS in different types of critically ill patients. Since the impairment of vascular endothelial cells, especially the glycocalyx damage, is deeply involved in the pathogenesis of CLS, they utilized a range of techniques, such as assay of serum biomarkers (angiopoietin-2, syndecan-1, intracellular adhesion molecule-1, lactate, and interleukin-6), intravital microscopic observation, and non-invasive bioelectrical impedance analysis. Ultimately, they found that a higher concentration of angiopoietin-2 was most significantly associated with the development of CLS [24]. Angiopoietin-2, stored and rapidly released upon stimulation from Weibel-Palade bodies in the endothelial cells, is known to bind endothelium-stabilizing receptor Tie2 and induce a rapid loss of glycocalyx [25]. Notably, CLS complicates not all but certain critically ill patients, particularly patients after cardiac surgery [26]. Therefore, identifying risk factors and determining the scoring system will help the anticipation and diagnosis of CLS in critically ill patients [27].

## Protection of glycocalyx

Protection and maintenance of the glycocalyx are critical in sepsis management. Although the current global guidelines do not propose specific treatments for glycocalyx protection [6], some recommendations are considered “friendly” to maintaining glycocalyx integrity. Preserving and restoring the glycocalyx helps to prevent vasoconstriction, tissue edema, and leukocyte/platelet adhesion, thus mitigating inflammation, thrombosis, and tissue hypoxia, which are all crucial aspects of sepsis management. The following part introduces a pragmatic approach to glycocalyx protection in sepsis management.

### Colloid substitution

A large amount of crystalloid resuscitation for sepsis and septic shock can increase the risk of heart failure and lung edema, which in turn are associated with increased mortality. Although albumin is more likely to maintain oncotic pressure than crystalloids, the superiority of albumin in terms of improved outcomes has not been confirmed by large-scale randomized controlled trials (RCTs) [28]. However, a recent prospective study in 50 patients with prolonged peripheral hypoperfusion showed that albumin infusion (20% 100 mL) improved tissue perfusion more than isotonic saline in resuscitated sepsis patients [29]. In SSCG 2021, “for adults with sepsis or septic shock, albumin use in patients who received large volumes of crystalloids over using crystalloids alone is suggested (Weak recommendation, low quality of evidence, Recommendation 34)” [6].

Albumin may protect the glycocalyx by carrying erythrocyte-derived S1P to the endothelium, which can mediate glycocalyx recovery by suppressing metalloproteinase activity [30]. However, clinical trial results were not supportive. In a phase 2 multicenter RCT in patients undergoing abdominal surgery, fluid administration included 20% albumin (100 mL) and dexamethasone, followed by 200 mL of 20% albumin with subsequent 1000 mL of crystalloid administered to the study group. The control group received crystalloids only. Glycocalyx injury, evaluated by syndecan-1 levels, did not differ between the groups [31]. Another post-hoc multicenter RCT analysis of albumin replacement in severe sepsis or septic shock (ALBIOS) reported a decrease of soluble VE-cadherin in the cohort receiving albumin but no significant changes in S1P and syndecan-1 [32].

Other than albumin, colloid therapy using fresh frozen plasma (FFP) has been proposed to protect the glycocalyx [33]. Diebel et al. [34] demonstrated a protective effect of plasma on the glycocalyx in a hypoxia/reoxygenation model in vitro. The exact mechanism is unknown but may represent the ability of fibrinogen/syndecan-1 to increase stress fibers [35]. Besides the above, plasma/FFP contains physiological oxidase, protease, and matrix metalloproteinase inhibitors that may help maintain the glycocalyx, and plasma/FFP decreases fluid requirements during resuscitation compared to crystalloids [36]. Recent studies report the beneficial effect of plasma exchange in severe sepsis and septic shock [37]. In an 80-patient pediatric trial of sepsis/septic shock in a non-overt DIC stage, early administration of FFP, low-dose heparin (LMWH), and tranexamic acid improved survival and prevented progression to overt disseminated intravascular coagulopathy (DIC), with no increased bleeding risk [38]. In another study of 31 septic shock patients, early plasma exchange reduced the imbalance between pro- and anticoagulant factors [39, 40]. Neither FFP nor plasma exchange is recommended in SSCG 2021 (Recommendation 60) [6]. However, these studies-of limited effectiveness-were published nearly at the same time as the guidelines.

In contrast to albumin, the protective effect of synthetic colloids has not been reported. An RCT of severe septic patients treated with hydroxyethyl starch (130/0.4) demonstrated an increased risk of death at day 90 [41]. In an animal model, 6% hydroxyethyl starch (130/0.4) provided protective effects on glycocalyx integrity and attenuated increased vascular permeability in sepsis [42]. In contrast, Kammer et al. examined the effect of 6% hydroxyethyl starch (130/0.4) and 5% albumin on coagulation and glycocalyx parameters in a post hoc analysis of an RCT performed in surgical patients [43]. Although patients treated with hydroxyethyl starch demonstrated greater abnormalities in thromboelastometry results, as compared to those treated with albumin, there were no significant differences in glycocalyx shedding, partial thromboplastin time, prothrombin time, and fibrinogen levels. In SSCG 2021, the use of starches or gelatin is not recommended either for the resuscitation of adults with sepsis or septic shock (Recommendations 35 and 36) [6].

### Catecholamine restriction

Vasoplegia, leading to refractory hypotension, is attributed to septic circulatory failure, which involves the massive production of vasodilators such as nitric oxide and prostaglandin [44]. Fluid resuscitation and catecholamine administration are the fundamental approaches to address this issue. However, when used clinically, very high doses of catecholamines are required for resuscitation in severe septic shock. Martin et al. [45] demonstrated increased glycocalyx shedding related to catecholamines in an in vitro model. Ostrowski et al. [46] examined the relationship between catecholamines and syndecan-1 in healthy volunteers subjected to endotoxin infusion and found that plasma catecholamine levels correlated positively with syndecan-1 levels. The degradation of the glycocalyx by catecholamines is explained by the increased activity of membrane-anchored proteins such as MMPs and ADAMs (a disintegrin and metalloproteinase). MMPs and ADAMs degrade the extracellular matrix, including the glycocalyx, and the activity of proteases is known to be increased by catecholamines, as they are in sepsis and septic shock. Endogenous norepinephrine levels have been correlated with plasma syndecan-1 levels; however, this has been in the setting of sepsis with concurrent thromboinflammation, which may cause direct glycocalyx damage [47].

DIC, limb ischemia, and acrocyanosis are common in patients with septic shock and shock liver. These patients are also vasoplegic, requiring high-dose vasopressor therapy. As a result, and despite high-dose norepinephrine at doses ranging from 0.58 − 4 mcg/kg/min, evidence of direct limb or digit ischemia or necrosis is uncommon in these critically ill patients, with a low frequency of 1.6−8%, and oftentimes no associated DIC or shock liver. Importantly, there is no direct literature supporting high-dose vasopressors as a primary cause of symmetrical peripheral gangrene or vascular injury independent of sepsis or septic shock [48].

In SSCG 2021, it is suggested that “for adults with septic shock on norepinephrine with inadequate mean arterial pressure levels, we suggest adding vasopressin instead of escalating the dose of norepinephrine. (Weak recommendation, moderate-quality evidence, Recommendation 38)” [6]. Adding vasopressin is usually considered when the norepinephrine dosing reaches 0.25−0.5 μg/kg/min, but this practice is not supported by robust scientific evidence. Similar to others, this recommendation was not made to protect the glycocalyx and aims to reduce the adverse effects of catecholamines, if possible, including impaired splanchnic and peripheral circulation [49, 50]. Hence, future studies should investigate the appropriateness of catecholamine dosage in clinical cases.

### Restrictive fluid therapy

Early fluid resuscitation is important to minimize the deleterious effects of tissue hypoperfusion. However, glycocalyx injury, as measured by elevated syndecan-1, is reported to be associated with the fluid volume required for resuscitation [51]. Although hypervolemia is potentially harmful to the glycocalyx, recent evidence suggests that excess fluids used to restore organ perfusion may damage vascular integrity and lead to organ dysfunction [52]. Previous studies suggested hypervolemia induces atrial natriuretic peptide release (ANP), leading to glycocalyx degradation [53]. ANP is released in response to atrial distention from vascular volume overload and has also been acknowledged as a potential endothelial glycocalyx sheddase. It is natural to think ANP partially counteracts volume overload by increasing endothelial microvascular permeability to facilitate fluid extravasation by endothelial glycocalyx degradation [54]. Jacob et al. [55] demonstrated a positive relationship between the effect of ANP and intravascular shedding of the glycocalyx in guinea pigs, and the effect was morphologically confirmed by electron microscopy. Taken together, fluid overload likely damages tissue circulation via glycocalyx damage; however, shock and sepsis are also important contributing factors. Byrne et al. [56] reported that fluid resuscitation resulted in a paradoxical increase in vasopressor requirement in an ovine model of endotoxemia and did not improve any of the microcirculatory or organ damage markers. However, extrapolation to clinical recommendations is uncertain.

An RCT examined a restricted resuscitation fluid protocol in 151 adult patients with septic shock and reported that restricted fluid therapy resulted in less frequent worsening of acute kidney injury and lower mortality [57]. However, a subsequent larger RCT with 1554 septic shock patients failed to show a better outcome with restricted intravenous fluid protocols [58]. Ultimately, the impact of restricted fluid therapy remains uncertain, especially in the setting of sepsis; still, based on published studies, it is important to consistently evaluate a patient’s response to fluid resuscitation. The SSCG 2021 reports there is insufficient evidence to recommend restrictive over liberal fluid strategies (Recommendation 45) [6]. Research on the effectiveness of restricted fluid therapy continues, with additional studies following the publication of JSSCG 2021. [58–60]. Although none demonstrate a benefit of restricted fluid therapy, a systematic review with meta-analysis concluded lower fluid volumes result in little to no difference in all-cause mortality compared with higher fluid volumes in adult patients with sepsis [61].

Another potential method to reduce fluid volume administration is to use hypertonic fluids. Smart et al. [62] reported no differences when evaluating 3.0% saline compared to isotonic saline to reduce syndecan-1 and hyaluronan in septic patients. However, it should be cautioned that hypernatremia can also cause glycocalyx degradation. Martin et al. [63] reported that exposure of human umbilical vein endothelial cells to hypoxia/reoxygenation and epinephrine, to mimic a shock-like insult, and subsequent treatment with a hypernatremic solution, leads to degradation of the endothelial glycocalyx. There is no recommendation regarding hypertonic solution in SSCG 2021.

### Corticosteroids

After decades of conflicting trial results, low-dose glucocorticoid administration is now recommended for the treatment of refractory shock. Per SSCG 2021, “For adults with septic shock and an ongoing requirement for vasopressor therapy, we suggest using IV corticosteroids” (Weak recommendation; moderate quality of evidence Recommendation 58) [6]. Glucocorticoids are recognized as anti-inflammatory agents, reducing proinflammatory cytokines and inflammatory mediators [64]. Consequently, they suppress leukocyte activation and may offer glycocalyx protection. In an animal model, Chappell et al. [65] demonstrated a preservative effect of hydrocortisone and antithrombin on the endothelial glycocalyx in a tumor necrosis factor-induced animal model, although protective effects have not been confirmed clinically. Pesonen et al. [66] performed an RCT in neonates following cardiac surgery and examined the effects of intraoperative administration of methylprednisolone (30 mg/kg). The study reported a reduction of syndecan-1 after cardiopulmonary bypass and 6 h postoperatively. By contrast, Yanase et al. [67] conducted a phase 2 RCT in patients undergoing major abdominal surgery and reported that intravenous dexamethasone (16 mg) and albumin administration did not reduce syndecan-1 postoperatively. Nevertheless, in critically ill COVID-19 patients, where glycocalyx injury is common, the beneficial effects of steroids secondary to ameliorating endothelial injury are recognized [68, 69].

### Anticoagulants

Thromboinflammation plays an important role in the progression of tissue injury in sepsis [20]. Although various trials have been performed to examine the effects of anticoagulant therapy, robust evidence supporting a beneficial role is lacking [70]. Anticoagulant therapy for sepsis is not discussed in SSCG 2021, and only low molecular weight heparin for thromboprophylaxis is recommended. “For adults with sepsis or septic shock, we recommend using LMWH over unfractionated heparin (UFH) for venous thromboembolism (VTE) prophylaxis (Strong recommendation, moderate quality of evidence, Recommendation 65)” [6]. Patients with sepsis are at risk for deep vein thrombosis (DVT) and pulmonary embolism (PE), and the incidence of VTE in critically ill patients is reportedly 4 to 15% [71]. Therefore, prevention of VTE by LMHW is important. Further, animal data suggest the protective properties of heparin on the glycocalyx [72]. Yini et al. [73] demonstrated reduced glycocalyx shedding related to UFH in a canine model of septic shock, and the effect of LMWH on glycocalyx shedding has been shown in a rat model [74]. However, the effects of heparins on organ damage in sepsis and in human patients are still uncertain.

Other than heparins, the protective effects of endogenous anticoagulants on glycocalyx integrity are reported [75]. Heparan sulfate is a glycosaminoglycan side-chain of the glycocalyx and acts as the cofactor for antithrombin. Antithrombin/heparan sulfate contributes to maintaining the antithrombotic property of the vascular lumen. Since antithrombin activity significantly decreases in sepsis, antithrombin repletion can modulate thromboinflammation in sepsis. Previous reports of antithrombin repletion in a sepsis model in rats suggest an ability to stabilize the glycocalyx by binding to vascular heparan sulfate [76]. Chappell et al. [77] also reported a similar effect of antithrombin in a guinea pig model of ischemia/reperfusion injury. Antithrombin, a serine protease inhibitor, can directly inhibit the serine protease thrombin that contributes to glycocalyx shedding. In Japanese sepsis guidelines, recombinant thrombomodulin is recommended for sepsis-associated disseminated intravascular coagulation [78]. In lipopolysaccharide-treated mice, pulmonary capillary injury was mitigated by recombinant thrombomodulin, consequently attenuating the damage in acute respiratory distress syndrome induced by endothelial injury [79].

### Glycocalyx component

Exogenously administered glycocalyx constituents such as hyaluronan could, theoretically, restore the glycocalyx structure, although these effects have not been confirmed even in animal models or clinically. Tenhunen et al. [80] examined whether exogenously administered hyaluronan counteracts intravascular volume depletion and maintains endothelial glycocalyx integrity in a porcine model of peritonitis. As a result, stroke volume variation, hemoconcentration, and plasma levels of syndecan-1 were comparable between the treatment and control groups. Sulodexide is a heparan sulfate-like compound resistant to degradation by heparanase. The protective effects of sulodexide are reported in a sepsis model of mice [81] and in children with septic shock [82]. Currently, there is no description of glycocalyx restoration in SSCG 2021; however, the use of sulodexide is recommended for the treatment of patients with COVID-19 [83].

### Glycemic control

Hyperglycemia is known to be associated to glycocalyx injury, and glycocalyx degradation has been reported in patients with diabetes [84]. In diabetic patients, glycemic control is associated with reductions in cardiovascular events [85]. In an acute inflammation model, hyperglycemia was reported to facilitate TNF-induced glycocalyx degradation in vitro [86]. Nieuwdorp et al. [87] reported that hyperglycemia increased plasma hyaluronan levels, endothelial dysfunction, and activation in coagulation in human subjects. The mechanism can be explained by the increased reactive oxygen species and receptor activation for advanced glycation end-products (RAGE). We also examined the effect of hyperglycemia in a rat model and demonstrated that neutrophil activation and NET formation were involved in glycocalyx injury [88]. In SSCG 2021, for adults with sepsis or septic shock, initiating insulin therapy at a glucose level of ≥ 180 mg/dL is recommended (Strong recommendation; moderate quality of evidence) [6]. However, since hypoglycemia is harmful to septic patients, glycemic control by intensive insulin therapy is not the current trend in sepsis management, and a typical target blood glucose range is set at 144–180 mg/dL (Recommendation 69) [6]. Future trials should consider targeting glycocalyx protection as part of achieving optimal glycemic control.

### Vitamin C

Vitamin C exhibits anti-inflammatory effects in sepsis as an antioxidant and as an essential substrate for neutrophil function [89]. An RCT enrolled 23 septic patients and showed thicker glycocalyx and a higher proportion of perfused capillaries by treatment with vitamin C [90]. Another post hoc study of the RCT examined the effect of high-dose vitamin C in sepsis-induced acute respiratory distress syndrome (CITRIS-ALI), demonstrating attenuated syndecan-1 by the treatment with vitamin C [91]. However, in the original RCT, vitamin C did not significantly improve organ function scores or markers of inflammation and vascular injury (e.g., soluble thrombomodulin) [92]. In addition, the latest RCT showed unexpected results; in adult patients with sepsis receiving vasopressors, intravenous vitamin C was associated with a higher risk of death and persistent organ dysfunction at 28 days compared to those who received placebo [93]. In SSCG 2021, the recommendation is “against the use of intravenous vitamin C” for adults with sepsis or septic shock (Weak recommendation, low quality of evidence, Recommendation 70) [6]. Thus, high-dose vitamin C may not be an appropriate choice for glycocalyx protection in sepsis.

## Conclusion

Given the critical role of the glycocalyx in maintaining vascular integrity, protecting this vital vascular component is an important consideration in sepsis management. In this review, we examined the role of the glycocalyx in sepsis, including current data regarding potential therapeutic modalities. While the recommendations in SSCG 2021 were not intended to protect the glycocalyx, some of the recommendations may be considered glycocalyx friendly. We propose that therapeutic strategies aiming at glycocalyx protection be considered in future sepsis studies and guidelines.

## Data Availability

Not applicable.

## Abbreviations

SSCG 2021: Surviving Sepsis Campaign Guidelines 2021
S1P: Sphingosine-1-phosphate
MMP: Matrix metalloproteinases
ICAM-1: Intercellular adhesion molecule-1
VCAM-1: Vascular cell adhesion molecule-1
NETs: Neutrophil extracellular traps
FFP: Fresh frozen plasma
LMWH: Low-dose heparin
ADAM: A disintegrin and metalloproteinase
ANP: Atrial natriuretic peptide release
UFH: Unfractionated heparin
VTE: Venous thromboembolism
DVT: Deep vein thrombosis
PE: Pulmonary embolism
RAGE: Receptor activation for advanced glycation end-products
